# Low neutrophil-to-lymphocyte ratio predicts overall survival benefit in advanced NSCLC patients with low PD-L1 expression and receiving chemoimmunotherapy

**DOI:** 10.3389/fonc.2023.1238876

**Published:** 2023-08-21

**Authors:** Chian-Wei Chen, Chien-Yu Lin, Jeng-Shiuan Tsai, Chia-Yin Lin, Chao-Chun Chang, Yi-Ting Yen, Yau-Lin Tseng, Po-Lan Su, Chien-Chung Lin

**Affiliations:** ^1^Department of Internal Medicine, National Cheng Kung University Hospital, College of Medicine, National Cheng Kung University, Tainan, Taiwan; ^2^Department of Medical Image, National Cheng Kung University Hospital, College of Medicine, National Cheng Kung University, Tainan, Taiwan; ^3^Department of Surgery, National Cheng Kung University Hospital, College of Medicine, National Cheng Kung University, Tainan, Taiwan; ^4^Department of Biomedical Engineering, College of Engineering, National Cheng Kung University, Tainan, Taiwan; ^5^Institute of Clinical Medicine, National Cheng Kung University Hospital, College of Medicine, National Cheng Kung University, Tainan, Taiwan; ^6^Department of Biochemistry and Molecular Biology, College of Medicine, National Cheng Kung University, Tainan, Taiwan

**Keywords:** neutrophil-to-lymphocyte ratio, non-small-cell lung cancer, chemotherapy, immune checkpoint inhibitors, survival

## Abstract

Although combination therapy including chemotherapy and immune checkpoint inhibitors (ICIs) improves overall survival (OS) of patients with non-small-cell lung cancer (NSCLC), there is a higher incidence of adverse events and treatment discontinuation. Since programmed death-ligand 1 (PD-L1) could not serve as a predictive biomarker, we investigated the neutrophil-to-lymphocyte ratio (NLR) as a predictive biomarker. In our previous research, we demonstrated that a low NLR could predict survival benefits when patients with high PD-L1 expression (> 50%) received chemoimmunotherapy as opposed to immunotherapy alone. In this current study, our objective is to evaluate this predictive capacity in patients with low PD-L1 expression (< 50%). A total of 142 patients were enrolled, 28 receiving combination therapy and 114 receiving chemotherapy alone. Progression-free survival (PFS) and OS were estimated using the Kaplan-Meier method and compared using the log-rank test. Patients who received combination therapy had significantly better PFS and OS than those who received monotherapy. In the subgroup of patients with low NLR, those who received combination therapy exhibited extended PFS and OS with clinical significance, which was also confirmed by multivariate Cox regression analysis. Our study demonstrates the potential use of NLR as a biomarker for predicting survival benefits when receiving combination therapy with chemotherapy and ICIs in patients with advanced NSCLC and low PD-L1 expression.

## 1 Introduction

Lung cancer is the leading cause of cancer-related death worldwide, and non-small-cell lung cancer (NSCLC) accounts for approximately 85% of all cases (1). Despite advancements in targeted therapy (2), nearly 50% of patients do not have detectable driver mutations and may benefit from the implementation of immune checkpoint inhibitors (ICI) (3). In contrast to patients with high PD-L1 expression (>50%) who exhibit an overall survival (OS) benefit from ICIs monotherapy (4–7), patients with low PD-L1 expression (< 50%) only obtain an OS benefit by receiving a combination of ICIs and chemotherapy (8, 9). However, combination therapy causes a higher incidence of grade 3 adverse events, which leads to approximately twice the risk of treatment discontinuation (8, 9). Moreover, the well-established PD-L1 biomarker could not identify the subgroup of patients with more clinical benefits from ICI addition in a pooled analysis (10). Considering this, it is of utmost importance to establish additional biomarkers that can accurately predict the treatment response.

The neutrophil-to-lymphocyte ratio (NLR) is an easily implemented and inexpensive biomarker that reflects the balance between pro-inflammatory neutrophils and antitumor lymphocytes in the systemic inflammatory response (11). Inflammation contributes to immune resistance in individuals with cancer. The cellular components of inflammation present in the tumor microenvironment can hinder adaptive immune responses and limit the effectiveness of anti-tumor treatments (12). Previous studies also demonstrated that the NLR could serve as a promising prognostic biomarker for patients with NSCLC undergoing treatment with ICIs (13). This presents an independent prognostic marker for overall survival, complementing PD-L1 (14). However, no study has yet focused on the predictive role of NLR. Recently, we also established that a low NLR could predict the progression-free survival (PFS) benefit in NSCLC patients with high PD-L1 expression who received combination therapy compared to those receiving only ICIs (15). We hypothesized that NLR could also be a predictive biomarker for NSCLC patients with PD-L1 expression below 50%. Therefore, we conducted a retrospective study to investigate its role in selecting the optimal patients for chemoimmunotherapy. We also explored the prognostic role of PD-L1 expression and its relationship with the NLR.

## 2 Materials and methods

### 2.1 Patients

This retrospective study was conducted at a tertiary referral center between January, 2018 and August, 2021 and enrolled patients with advanced NSCLC who received first-line therapy. Patients with oncogenic driver mutations and PD-L1 tumor proportion score greater than 50%, as determined by the Dako PD-L1 22C3 pharmDx™ kit, were excluded. For complete staging of the patients, chest computed tomography (CT), brain magnetic resonance imaging (MRI), and whole-body bone scans were performed following the tumor, node, and metastasis (TNM) classification system proposed by the 8th edition of the American Joint Committee on Cancer. The decision to administer chemotherapy alone or in combination with ICIs was at the discretion of the treating physician. Baseline patient characteristics were documented, including age, sex, histological subtype, TNM stage, smoking status, Eastern Cooperative Oncology Group (ECOG) performance status (PS), and presence of brain or liver metastases. Comprehensive details regarding the combined therapy and chemotherapy are presented in **Supplementary Table S1**. During the treatment period, blood test data prior to the first and third courses of chemotherapy were recorded to calculate the Neutrophil-to-Lymphocyte Ratio (NLR), representing pre-treatment and post-treatment NLR respectively. Blood tests were conducted within one week prior to the initiation of treatment. All data were de-identified in accordance with approved protocols and principles of the Declaration of Helsinki. This study was approved by the Institutional Review Board of National Cheng Kung University Hospital (IRB number: B-ER-109-344).

### 2.2 PFS and OS analysis

Following the initiation of treatment, patients underwent chest CT every 12 weeks to assess the tumor response. OS was calculated from the start of treatment until the date of death, whereas PFS was determined from the date of treatment initiation until the date of radiological progression, discontinuation due to adverse events, or death, based on the Response Evaluation Criteria in Solid Tumors version 1.1 (16). For patients who did not experience disease progression, censoring was performed based on the date of the last follow-up. The study carried out a subgroup survival analysis, wherein patients were categorized based on the median value of the NLR. PFS and OS were compared within each subgroup.

### 2.3 Statistical analysis

The frequencies and descriptive statistics of the demographic and clinical variables were calculated. All variables analyzed in this study were categorical and were compared using either the chi-square test or Fisher’s exact test. The Kaplan-Meier method was employed to estimate both the PFS and OS of all patients, which were then compared using the log-rank test. COX proportional hazard regression analysis was performed to identify independent prognostic factors. The selection of factors to predict and determine survival outcomes was based on previous studies that investigated prognostic factors (17). Statistical analyses were conducted using SAS version 9.4 (SAS Institute, Cary, NC, USA). All p-values reported in this study were two-sided, and p< 0.05 was considered statistically significant.

## 3 Results

### 3.1 Patient characteristics

**Figure 1:**
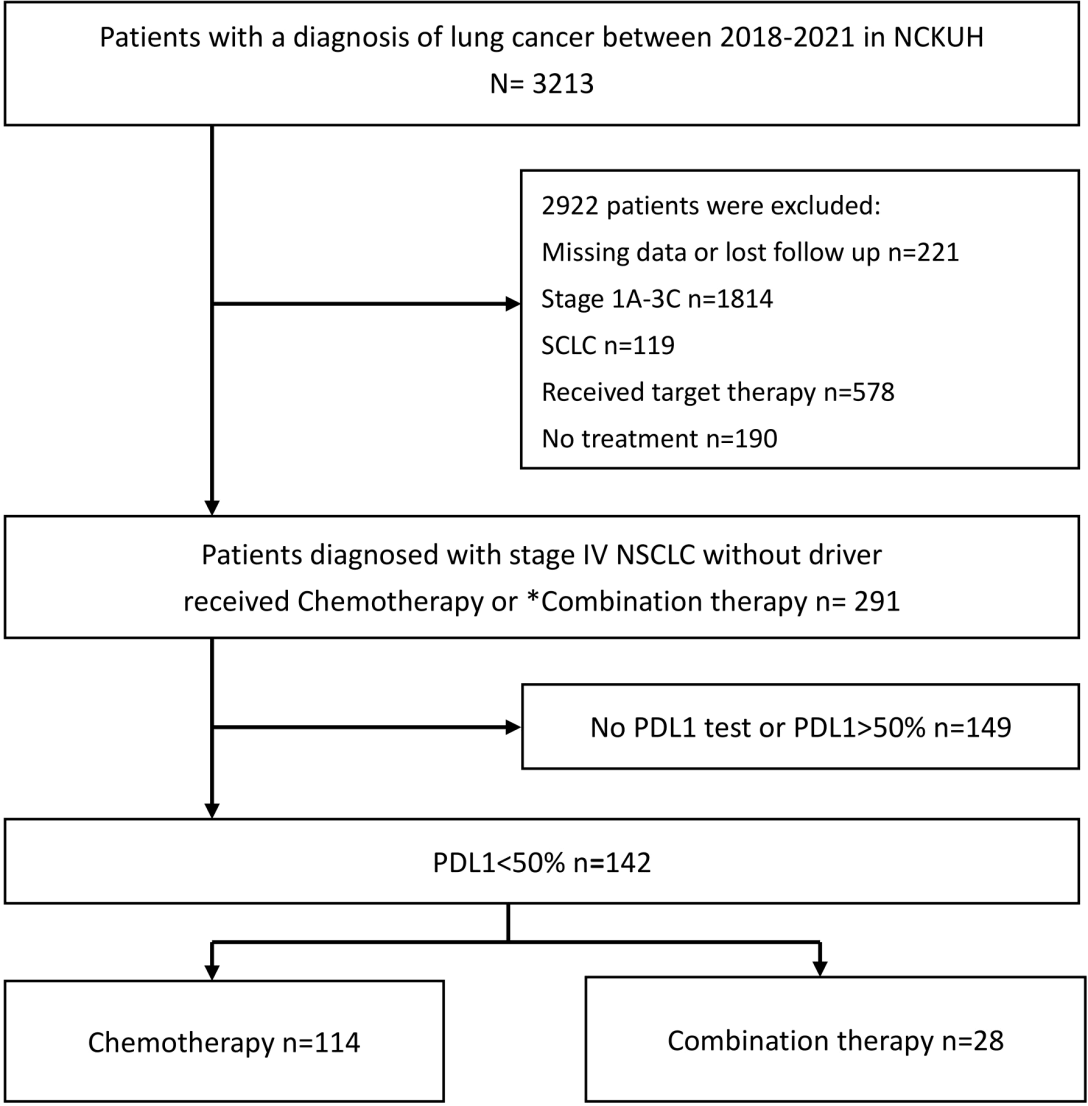
Flowchart outlining the patient recruitment process for the study. NCKUH, National cheng kung university hospital; NSCLC, non-small cell lung cancer; PD-L1, programmed death ligand-1; SCLC, small cell lung cancer; ICI, immune checkpoint inhibitor. *Combination therapy: Chemotherapy combined with ICIs.

A total of 142 participants were enrolled in the present study, of whom 114 received chemotherapy alone, and 28 received combination therapy with chemotherapy and ICIs. The detailed patient enrollment process is shown in **Figure 1**. The PFS and OS of all participants were analyzed. **Table 1** summarizes the baseline characteristics of all patients, indicating similarities between the monotherapy and combination therapy groups, except for the proportion of patients with PD-L1 < 1%. All the participants had advanced NSCLC. The median patient age was 67 years (interquartile range,59–75 years), and there were 113 men (79.5%) and 29 women (20.4%). Histologically, nonsquamous cancer types were observed in 103 patients (72.5%), whereas squamous types were observed in 39 patients (27.4%). Additionally, 42 patients (29.6%) had brain metastases and 15 (10.6%) had liver metastases.

**Table 1:** Baseline characteristics.

Variable	Combination therapy (n=28)	Chemotherapy (n=114)	p-values
Age (years), median (interquartile range)	64 (57.5-72.3)	67 (60.0-76.8)	0.594
Male sex, n (%)	22 (79)	91(80)	0.883
PD-L1<1%, n (%)	6(21)	53(46)	0.016
Histologic subtype, n (%)			0.204
Non-squamous NSCLC	23(82)	80(70)	
Squamous cell carcinoma	5(18)	34(30)	
Stage, n (%)			0.442
4A	17(61)	60(53)	
4B	11(40)	54(47)	
ECOG PS >2, n (%)	2(7)	12(11)	0.590
Smoking, n (%)	16(57)	72(63)	0.557
Brain metastasis, n (%)	6(21)	36(32)	0.292
Liver metastasis, n (%)	2(7)	13(11)	0.511

ECOG, Eastern Cooperative Oncology Group; NSCLC, non-small cell lung cancer PD-L1, programmed death ligand-1; PS, performance status.

### 3.2 Progression-free survival and overall survival

**Figure 2:**
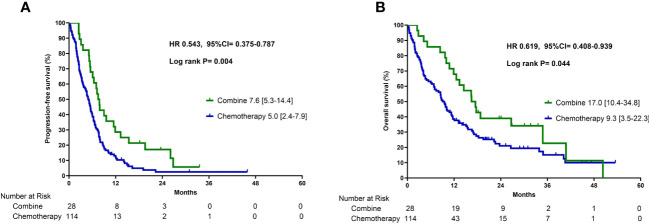
Progression-free survival **(A)** and overall survival **(B)** in patients who received combination therapy or chemotherapy alone.

Patients who received combination therapy demonstrated a significantly longer median PFS of 7.6 months (interquartile range [IQR] 5.3–14.4) than patients who received chemotherapy monotherapy (5.0 months, IQR 2.4–7.9) (p =0.004, **Figure 2A**). The median OS for patients who received combination therapy was 17.0 months (IQR 10.4–34.8), which was also significantly longer than that of patients who received chemotherapy monotherapy (9.3 months, IQR 3.5–22.3) (p =0.044, **Figure 2B**). Multivariate Cox proportional hazards regression analysis was performed to identify independent prognostic factors. The results indicated that combination therapy with chemotherapy and ICIs was an independent prognostic factor for PFS with a hazard ratio (HR) of 0.56 (95% CI:0.36–0.88, p =0.011) and OS with a HR of 0.60 (95% CI:0.37–0.99, p =0.045) (**Table 2**). The good performance status was an independent prognostic factor for both PFS and OS (**Table 2**).

**Table 2:** Multivariable Cox proportional hazard regression analysis to identify the prognostic factors in PFS and OS.

	HR for PFS (95% CI)	p-value	HR for OS (95% CI)	p-value
Age (Age<65 vs. ≥65 y/o)	0.980 (0.684-1.404)	0.9115	0.661 (0.443-0.987)	0.0429
Sex (Female vs. Male)	0.900 (0.501-1.617)	0.7240	0.512 (0.265-0.990)	0.0467
Stage (4A vs. 4B)	0.533 (0.36-0.789)	0.0017	0.674 (0.444-1.023)	0.0640
PDL1 (PDL1 <1% vs. PDL1>1%)	0.931 (0.641-1.351)	0.7059	0.801 (0.532-1.206)	0.2886
Histology (Non-squamous vs. squamous NSCLC)	0.681 (0.449-1.032)	0.0702	0.699 (0.450-1.085)	0.1102
Liver metastasis (absence vs. presence)	1.017 (0.568-1.82)	0.9555	0.875 (0.478-1.599)	0.6635
Brain metastasis (absence vs. presence)	1.247 (0.816-1.907)	0.3082	1.145 (0.727-1.805)	0.5589
Treatment (Combination therapy vs. Chemotherapy)	0.509 (0.321-0.806)	0.0040	0.603 (0.367-0.988)	0.0447
Smoking (non-smoker vs. Smoker)	1.141 (0.702-1.854)	0.5936	1.098 (0.658-1.832)	0.7210
ECOG (≤1 vs. ≥2)	0.328 (0.178-0.605)	0.0003	0.436 (0.242-0.783)	0.0055

ECOG, Eastern Cooperative Oncology Group; HR, hazard ratio; NSCLC, non-small cell lung cancer; OS, overall survival; PD-L1, programmed death ligand-1; PFS, progression-free survival; PS, performance status.

### 3.3 Subgroup analysis

**Figure 3:**
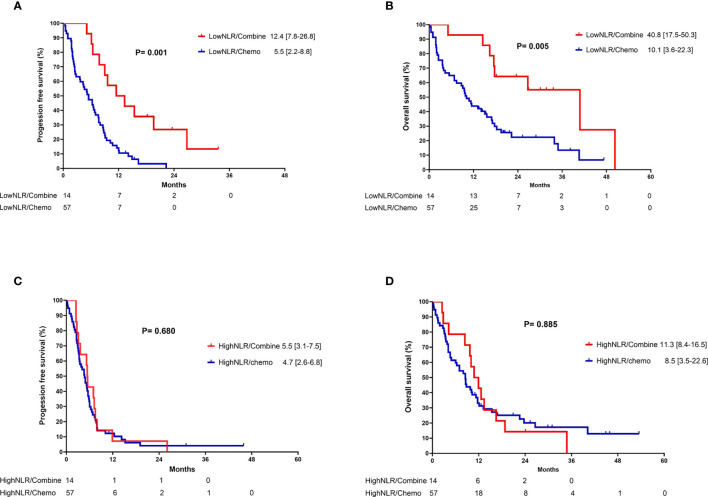
Kaplan-Meier curves of **(A)** progression-free survival and **(B)** overall survival for patients with low neutrophil-to-lymphocyte ratio who received either combination therapy or monotherapy; **(C)** progression-free survival and **(D)** overall survival for patients with high neutrophil-to-lymphocyte ratio who received either combination therapy or monotherapy.

**Figure 4:**
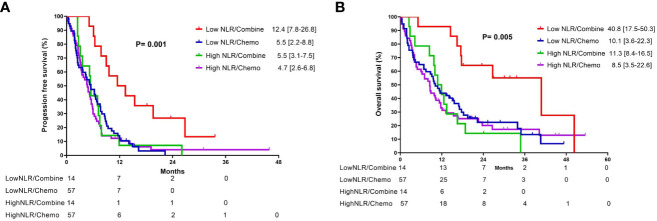
Kaplan-Meier curves of **(A)** progression-free survival and **(B)** overall survival for patients with different neutrophil-to-lymphocyte ratio who received either combination therapy or monotherapy.

In the subgroup analysis, patients were classified into two groups based on the pre-treatment neutrophil-to-lymphocyte ratio (NLR): high NLR group (NLR ≥ 3.7) and low NLR group (NLR < 3.7). For patients in the low pre-treatment NLR subgroup, those who received combination therapy had significantly longer PFS (12.4 vs. 5.5 months, p =0.001) and OS (40.8 vs. 10.1 months, p = 0.005) compared to those who received chemotherapy alone (**Figures 3A, B**). In contrast, among patients with a high pre-treatment NLR, similar PFS and OS rates were observed between those who received combination therapy and those who received chemotherapy alone (**Figures 3C, D**). In addition, the survival outcome was comparable between individuals with a low pre-treatment NLR who received chemotherapy alone and those with a high pre-treatment NLR (chemotherapy and combination) (**Figures 4A, B**). In order to identify the independent prognostic factor among subgroup patients with low NLR, we used Cox proportional hazards regression analysis and found that the combination therapy was still an independent predictor for both PFS (HR 0.22, 95% CI:0.10–0.47, p < 0.0001) and OS (HR 0.22, 95% CI:0.09–0.54, p =0.001) compared to chemotherapy alone (**Table 3**). Similarly, a good performance status was also a good prognostic factor (**Table 3**).

**Table 3:** Multivariable Cox proportional hazard regression analysis to identify the prognostic factors in PFS and OS in the subgroup of patients with low NLR (< 3.7).

	HR for PFS (95% CI)	p-value	HR for OS (95% CI)	p-value
Age (Age <65 vs. ≥65 y/o)	0.908 (0.525-1.569)	0.7284	0.486 (0.25-0.945)	0.0334
Sex (Female vs. Male)	0.769 (0.310-1.909)	0.5713	0.334 (0.106-1.055)	0.0618
Stage (4A vs. 4B)	0.521 (0.274-0.99)	0.0465	0.658 (0.335-1.292)	0.2237
PDL1 (PD-L1 <1% vs. PD-L1 >1%)	1.425 (0.827-2.456)	0.2026	0.761 (0.406-1.425)	0.3935
Histology (Non-squamous vs. squamous NSCLC)	0.635 (0.325-1.239)	0.1828	0.398 (0.194-0.817)	0.0120
Liver metastasis (absence vs. presence)	1.096 (0.448-2.683)	0.8407	0.696 (0.272-1.779)	0.4490
Brain metastasis (absence vs. presence)	0.895 (0.433-1.852)	0.7655	1.071 (0.509-2.253)	0.8556
Treatment (Combination therapy vs. Chemotherapy)	0.215 (0.099-0.466)	<.0001	0.219 (0.089-0.543)	0.0010
Smoking (Smoker vs. non-smoker)	1.094 (0.487-2.455)	0.8283	1.188 (0.468-3.013)	0.7173
ECOG (≤1 vs. ≥2)	0.125 (0.045-0.347)	<.0001	0.141 (0.049-0.406)	0.0003

ECOG, Eastern Cooperative Oncology Group; HR, hazard ratio; NSCLC, non-small cell lung cancer; OS, overall survival; PD-L1, programmed death ligand-1; PFS, progression-free survival; PS, performance status.

To compare the predictive power of PD-L1 and NLR, we assessed the progression-free survival (PFS) and overall survival (OS) in patients who received different treatment strategies. These patients were categorized based on NLR (with a cut-off value of 3.7, **Supplementary Figures 1A, B**) and PD-L1 (with a cut-off value of 1%, **Supplementary Figures 1C, D**). Although there was a trend suggesting that a PD-L1 value of less than 1% could predict better overall survival with chemoimmunotherapy, the difference did not reach statistical significance. However, a low NLR was a significant predictor of survival benefit when receiving chemoimmunotherapy. We also conducted a Spearman correlation analysis to investigate the relationship between pre-treatment NLR (preNLR), NLR at six weeks post-treatment initiation (postNLR), dynamic NLR (dynamicNLR), and PD-L1 expression. However, none of these correlations were statistically significant. (**Supplementary Figure 2**).

To further confirm the predictive power of NLR, we conducted a subgroup analysis on PFS (**Supplementary Figure 3**) and OS (**Supplementary Figure 4**), stratified based on PD-L1 expression levels below 10% and 5%. This analysis revealed that combination therapy did not significantly improve PFS and OS compared to chemotherapy alone for patients with PD-L1 expression levels below 10% (**Supplementary Figures 3A, 4A**) and 5% (**Supplementary Figure 3D, 4D**). However, when these patients were further stratified by NLR, those with an NLR <3.7 demonstrated improved PFS (**Supplementary Figures 3B, E**) and OS (**Supplementary Figures 4B, E**) upon receiving chemoimmunotherapy, while those with an NLR >3.7 showed no difference. In a similar vein, NLR could also predict the PFS (**Supplementary Figure 5**) and OS (**Supplementary Figure 6**) benefits among patients with PD-L1 expression 10-50% or 5-50%.

## 4 Discussion

Our findings suggest that combination therapy with chemotherapy and ICIs could provide significant PFS and OS benefits in patients with advanced NSCLC and low PD-L1 expression (< 50%). In the subpopulation with a pretreatment low NLR, combination therapy with chemotherapy and ICIs resulted in extended PFS and OS benefits, whereas no difference was observed in PFS and OS among patients with a high pretreatment NLR. This result suggests that among NSCLC patients with low PD-L1 expression, NLR could be used to predict the survival benefit of adding ICIs.

Treatment strategies for NSCLC patients with low PD-L1 expression remain challenging. Previous phase 3 trials demonstrated that ICIs monotherapy did not provide an OS benefit in this subgroup of patients (6, 18). Nonetheless, subsequent clinical trials investigating the treatment efficacy of combining chemotherapy and ICIs have provided promising results (5, 8, 19, 20) and treatment guidelines recommend this strategy (21). Despite the higher response rate and longer overall survival, the risks of adverse events and treatment discontinuation also increased. In addition, according to a study that investigated the benefit of combining chemotherapy and pembrolizumab (5, 8), the HRs of OR were almost the same across all PD-L1 expression levels, indicating that additional biomarkers are needed to predict the clinical benefit of adding ICIs.

The composition of tumor immune microenvironment (TME), including various immune cell types, vascular structures, and signaling molecules, is associated with treatment response to immunotherapy (22). The enrichment of cytotoxic T lymphocytes in the TME leads to cytokine release, which subsequently inhibits tumor growth (23). In contrast, the accumulation of neutrophils is usually associated with pro-tumor inflammation via inhibition of the T lymphocyte cytotoxic activity (24). Although these markers are promising, they are difficult to implement clinically because of their high costs and time-consuming analysis process (25).

To better predict the clinical benefit of immunotherapy, the surrogate biomarker blood NLR has been increasingly studied. In a retrospective study that enrolled patients with early stage NSCLC who underwent surgery, those with a more advanced stage cancer had a higher NLR (26). Patients with stage I NSCLC and preoperative NLR > 2.5 had a significantly reduced 5-year overall survival (27), with similar results observed among patients with locally advanced NSCLC (stage IIIA and IIIB) (28, 29). In addition to being a prognostic biomarker for the survival of patients with early stage NSCLC, the NLR was also investigated as a prognostic biomarker for patients who received immunotherapy. In a cohort study conducted by Mezquita et al., patients with a pre-treatment derived NLR > 3 and lactate dehydrogenase levels > upper limit of normal had significantly shorter OS when receiving immunotherapy (30). Similar results were also found in studies focusing on patients who receiving chemoimmunotherapy (31). However, all the studies mentioned above utilize NLR as prognostic biomarkers, not as predictive ones. To identify subgroups of patients who would potentially benefit from immunotherapy, we subdivided the patients based on different NLR values. This allowed us to compare the effectiveness of chemotherapy and immunotherapy in each subgroup. In our previous study, which enrolled patients with high PD-L1 expression, a low NLR was found to predict improved PFS in patients receiving a combination of chemotherapy and pembrolizumab compared to those receiving pembrolizumab monotherapy (15). In this study, we further established that patients with a low NLR significantly benefited from improved PFS and OS when undergoing chemoimmunotherapy compared to chemotherapy alone. This finding underscores the predictive utility of the NLR in favoring chemoimmunotherapy.

The present study has limitations. First, this was a single-center retrospective study, and the limited number of cases precluded definitive conclusions. A prospective study is warranted to further validate the predictive value of the NLR among NSCLC patients with low PD-L1 expression. Second, the baseline characteristics were imbalanced between patients who received combination therapy and those who received chemotherapy alone. However, we performed a multivariate analysis to adjust for potential confounding factors. Third, underlying immunotherapy-related genomic alterations, including those in *KRAS, STK11*, and *KEAP1*, were not assessed. Even though the predictive role of genomic alterations in immunotherapy remains controversial (32), their prognostic role should be investigated in future studies by TME assessments and comprehensive genomic profiling.

In conclusion, we demonstrated that a low NLR could predict survival benefits when receiving chemoimmunotherapy in NSCLC patients with low PD-L1 expression. Nonetheless, prospective studies are required to validate these findings.

## Data availability statement

The raw data supporting the conclusions of this article will be made available by the authors, without undue reservation.

## Ethics statement

The studies involving humans were approved by the Institutional Review Board of National Cheng Kung University Hospital. The studies were conducted in accordance with the local legislation and institutional requirements. The ethics committee/institutional review board waived the requirement of written informed consent for participation from the participants or the participants’ legal guardians/next of kin because of the retrospective nature of present study.

## Author contributions

Conceptualization: C-WC, Chien-YL, J-ST, P-LS, and C-CL. Methodology: C-WC, Chien-YL, J-ST, P-LS, and C-CL. Formal analysis: Chien-YL and P-LS. Investigation: Chien-YL, C-WC and P-LS. Resources: P-LS and C-CL. Data curation: Chien-YL, C-WC, J-ST, Chia-YL, C-CC, Y-TY, and Y-LT. Writing—original draft preparation: Chien-YL and C-WC. Writing—review and editing: P-LS and C-CL. Project administration: P-LS and C-CL. Funding acquisition: Chien-YL, P-LS, and C-CL. All authors have read and agreed to the published version of the manuscript.
