# Supplementary material for: Low neutrophil-to-lymphocyte ratio predicts overall survival benefit in advanced NSCLC patients with low PD-L1 expression and receiving chemoimmunotherapy
Source: Front Oncol. 2023 Aug 21;13:1238876. doi: 10.3389/fonc.2023.1238876 (PMC10475717; doi:10.3389/fonc.2023.1238876)
Supplement: Supplementary file 1 [file DataSheet_1.pdf]

Supplementary Table 1. Detailed regimen

**Combination therapy**	n	**Chemotherapy**	n
Pembrolizumab /Carboplatin/Pemetrexed	14	Cisplatin/Pemetrexed	40
Pembrolizumab /Cisplatin/Pemetrexed	8	Carboplatin/Pemetrexed	26
Pembrolizumab /Carboplatin/Paclitaxel	4	Cisplatin/ Gemcitabine	15
Pembrolizumab/ Pemetrexed	1	Navelbine	11
Pembrolizumab /Cisplatin/Paclitaxel	1	Pemetrexed	6
		Gemcitabine	6
		Cisplatin/Docetaxel	4
		Carboplatin/Gemcitabine	3
		Docetaxel	2
		Carboplatin/Docetaxel	1

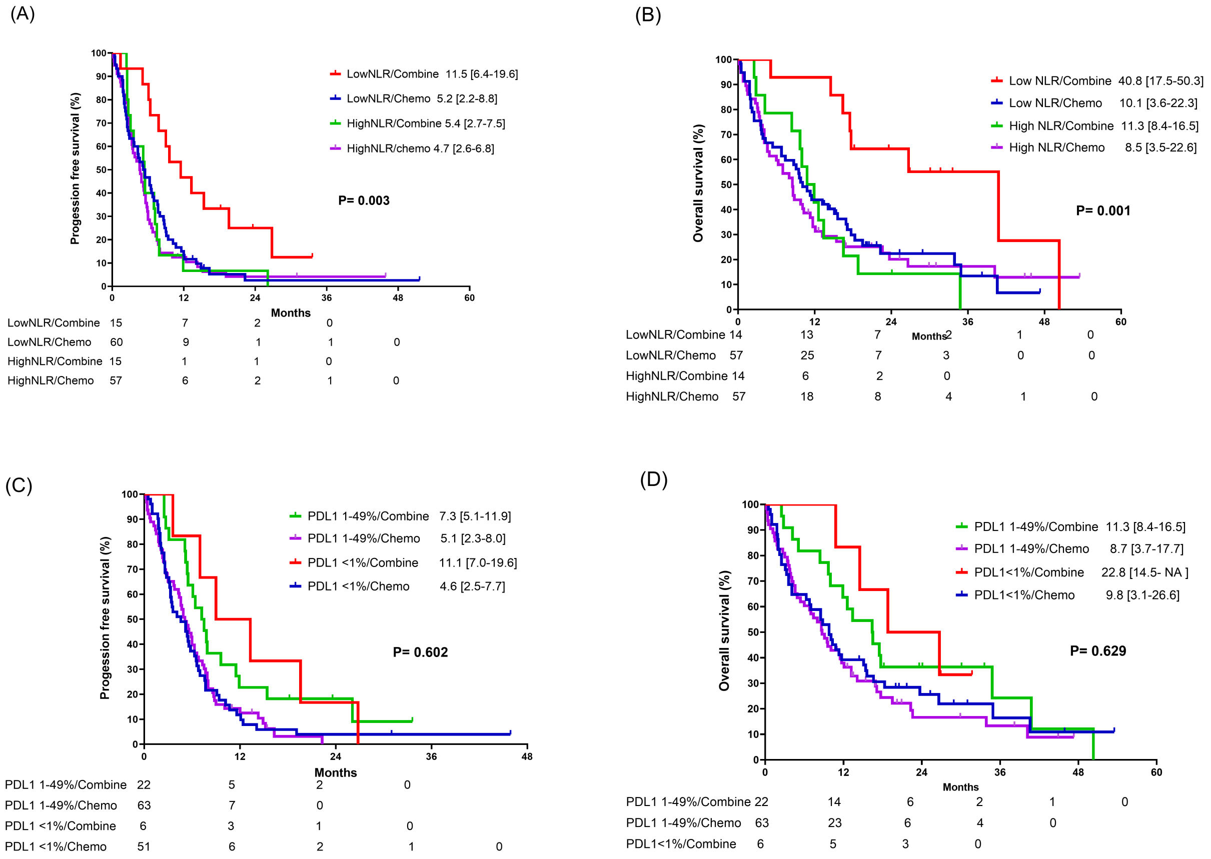

Supplementary Figure 1. Kaplan-Meier curves of progression-free survival and overall survival with different neutrophil-to-lymphocyte ratio or PD-L1 tumor proportion score, who received either combination therapy or monotherapy.

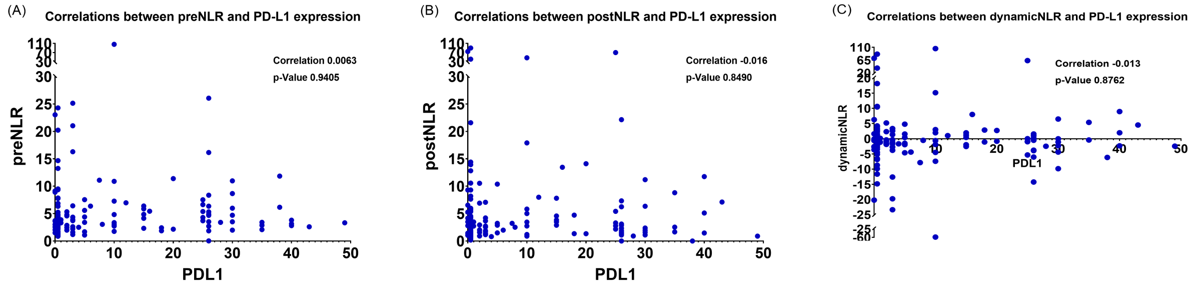

Supplementary Figure 2. Spearman correlation between PD-L1 and (A) pre-treatment NLR, (B) post-treatment NLR, and (C) change of NLR.

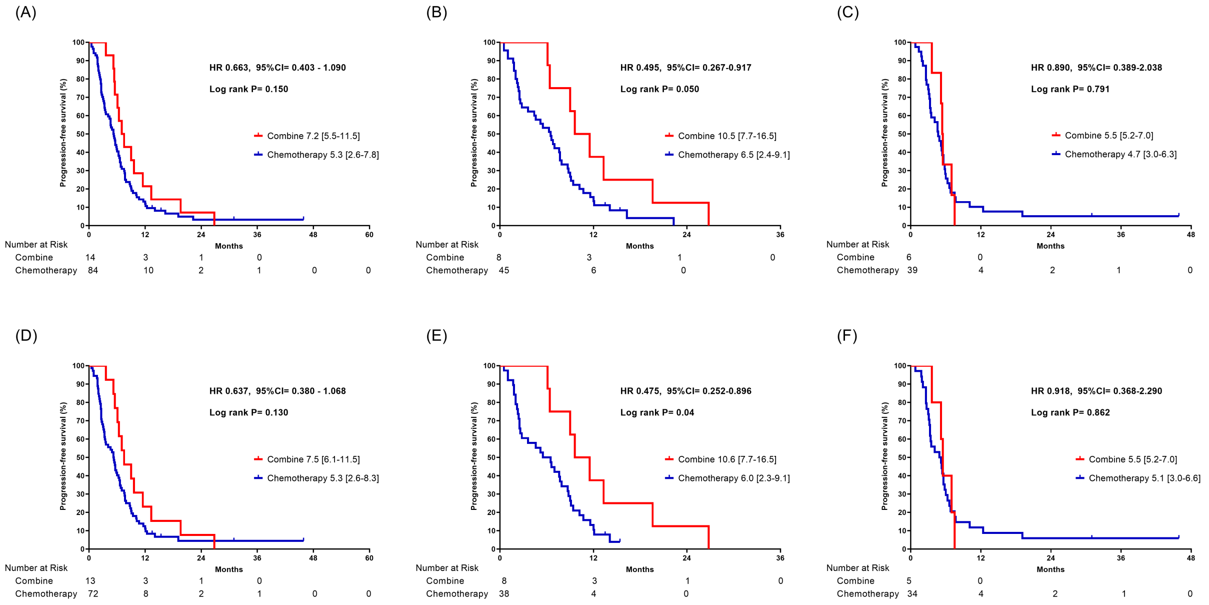

Supplementary Figure 3. Kaplan-Meier curves show progression-free survival for patients with PD-L1 expression <10% (A), and those further stratified by low (B) and high (C) neutrophil-to-lymphocyte ratios. The same applies for patients with PD-L1 expression <5% (D), further stratified by low (E) and high (F) ratios.

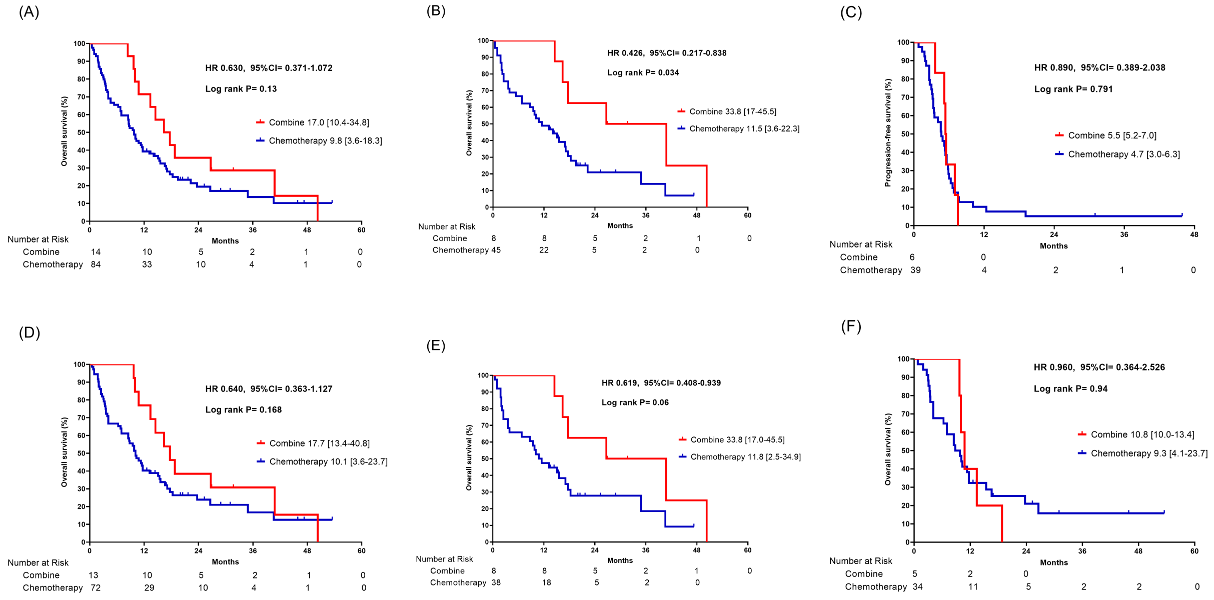

Supplementary Figure 4. Kaplan-Meier curves show overall survival for patients with PD-L1 expression <10% (A), and those further stratified by low (B) and high (C) neutrophil-to-lymphocyte ratios. The same applies for patients with PD-L1 expression <5% (D), further stratified by low (E) and high (F) ratios.

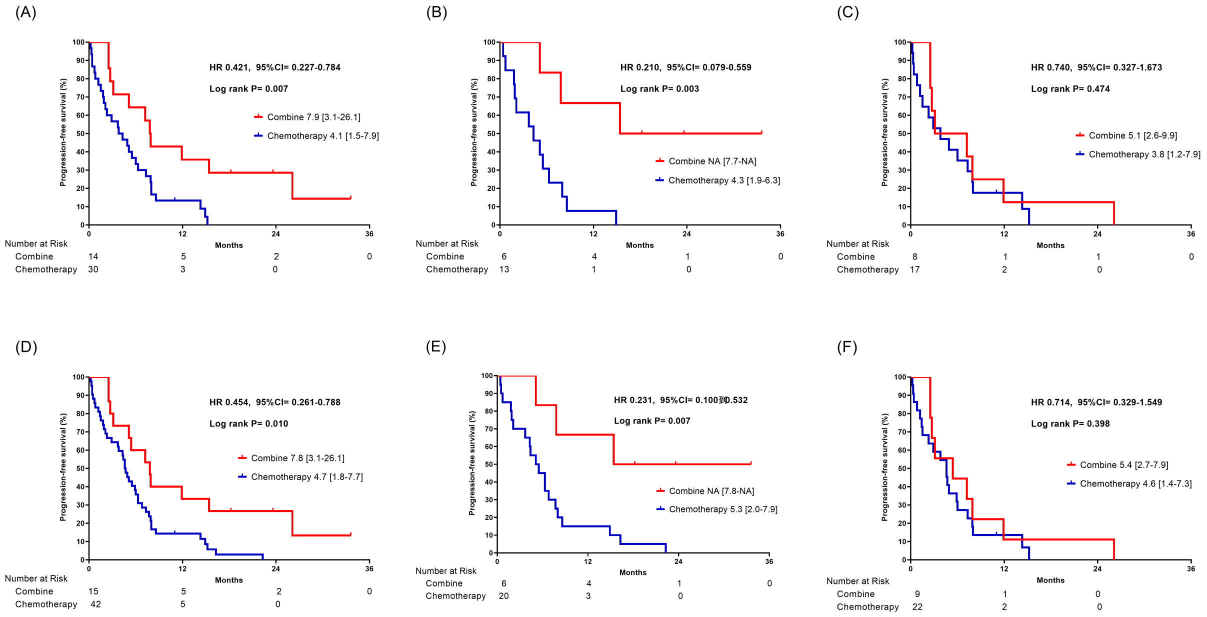

Supplementary Figure 5. Kaplan-Meier curves show progression-free survival for patients with PD-L1 expression 10-50% (A), and those further stratified by low (B) and high (C) neutrophil-to-lymphocyte ratios. The same applies for patients with PD-L1 expression 5-50% (D), further stratified by low (E) and high (F) ratios.

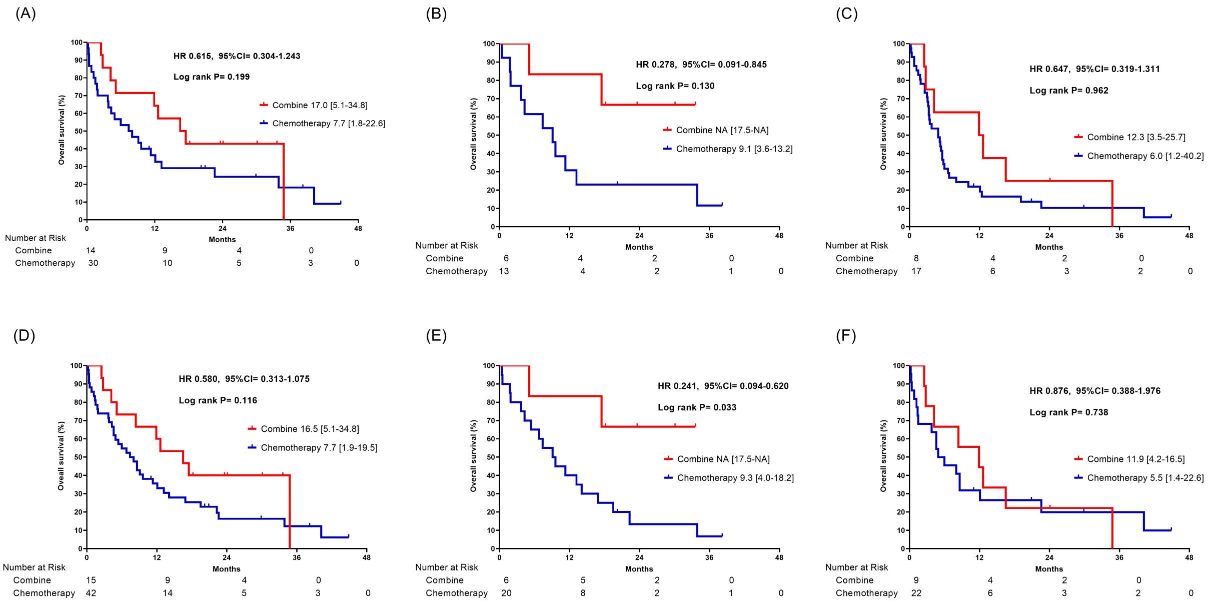

Supplementary Figure 6. Kaplan-Meier curves show overall survival for patients with PD-L1 expression 10-50% (A), and those further stratified by low (B) and high (C) neutrophil-to-lymphocyte ratios. The same applies for patients with PD-L1 expression 5-50% (D), further stratified by low (E) and high (F) ratios.
